# Supplementary material for: Ancient Evolutionary History of Human Papillomavirus Type 16, 18 and 58 Variants Prevalent Exclusively in Japan
Source: Viruses. 2022 Feb 24;14(3):464. doi: 10.3390/v14030464 (PMC8953638; doi:10.3390/v14030464)
Supplement: Supplementary file 1 [file viruses-14-00464-s001.zip › Tanaka_viruses_Figure S1.pdf]

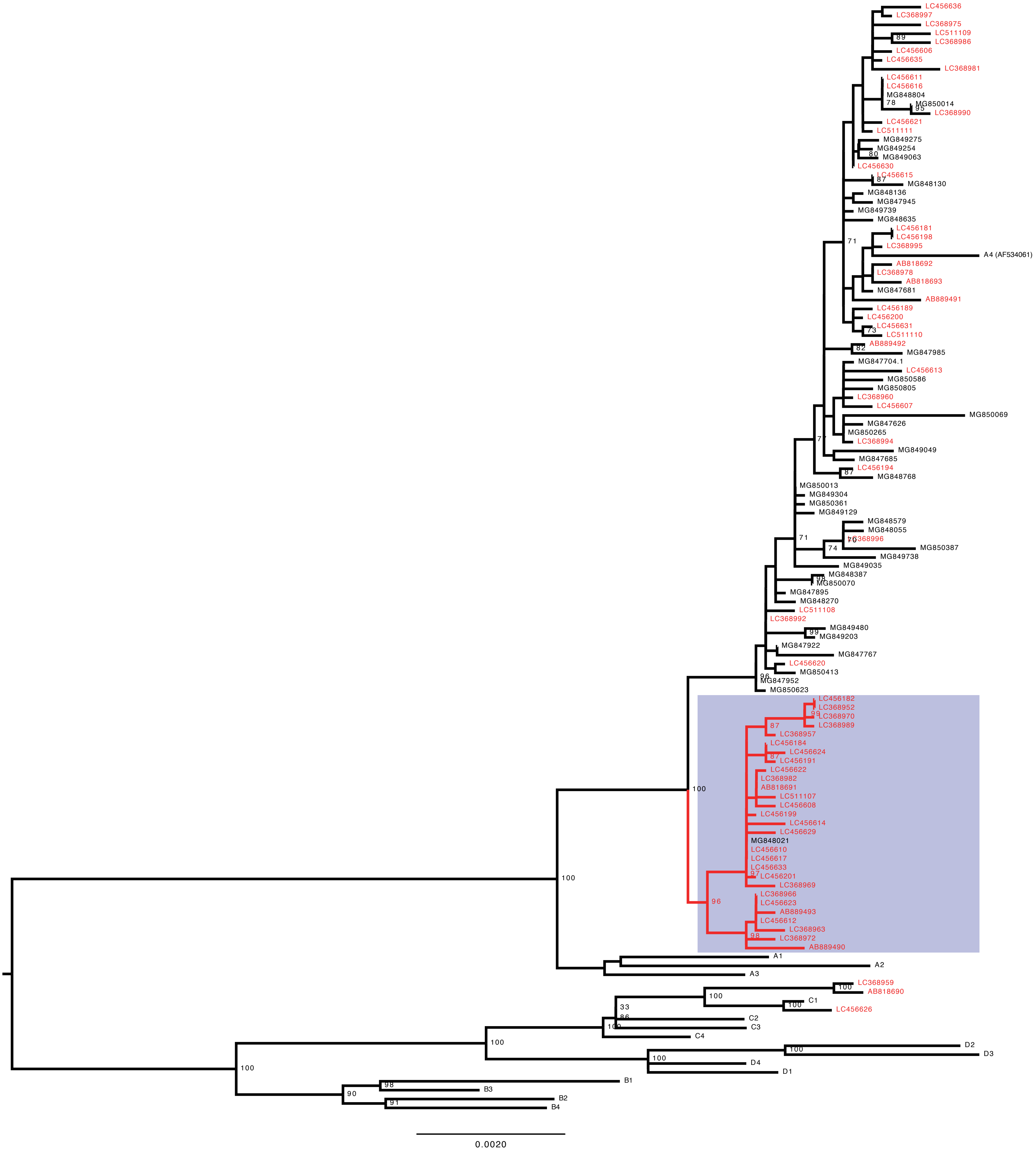

Figure S1. Maximum likelihood phylogenetic tree of HPV16 genomes including 42 A4 genomes from Mirabello et al. Bootstrap values >70% are displayed. The red taxa indicate A4 genomes from Japan. Purple area marks the Japan-specific cluster of the sublineage A4. Scale bar, nucleotide substitutions per site.
