# Supplementary material for: Ancient Evolutionary History of Human Papillomavirus Type 16, 18 and 58 Variants Prevalent Exclusively in Japan
Source: Viruses. 2022 Feb 24;14(3):464. doi: 10.3390/v14030464 (PMC8953638; doi:10.3390/v14030464)
Supplement: Supplementary file 1 [file viruses-14-00464-s001.zip › Tanaka_viruses_Figure S2.pdf]

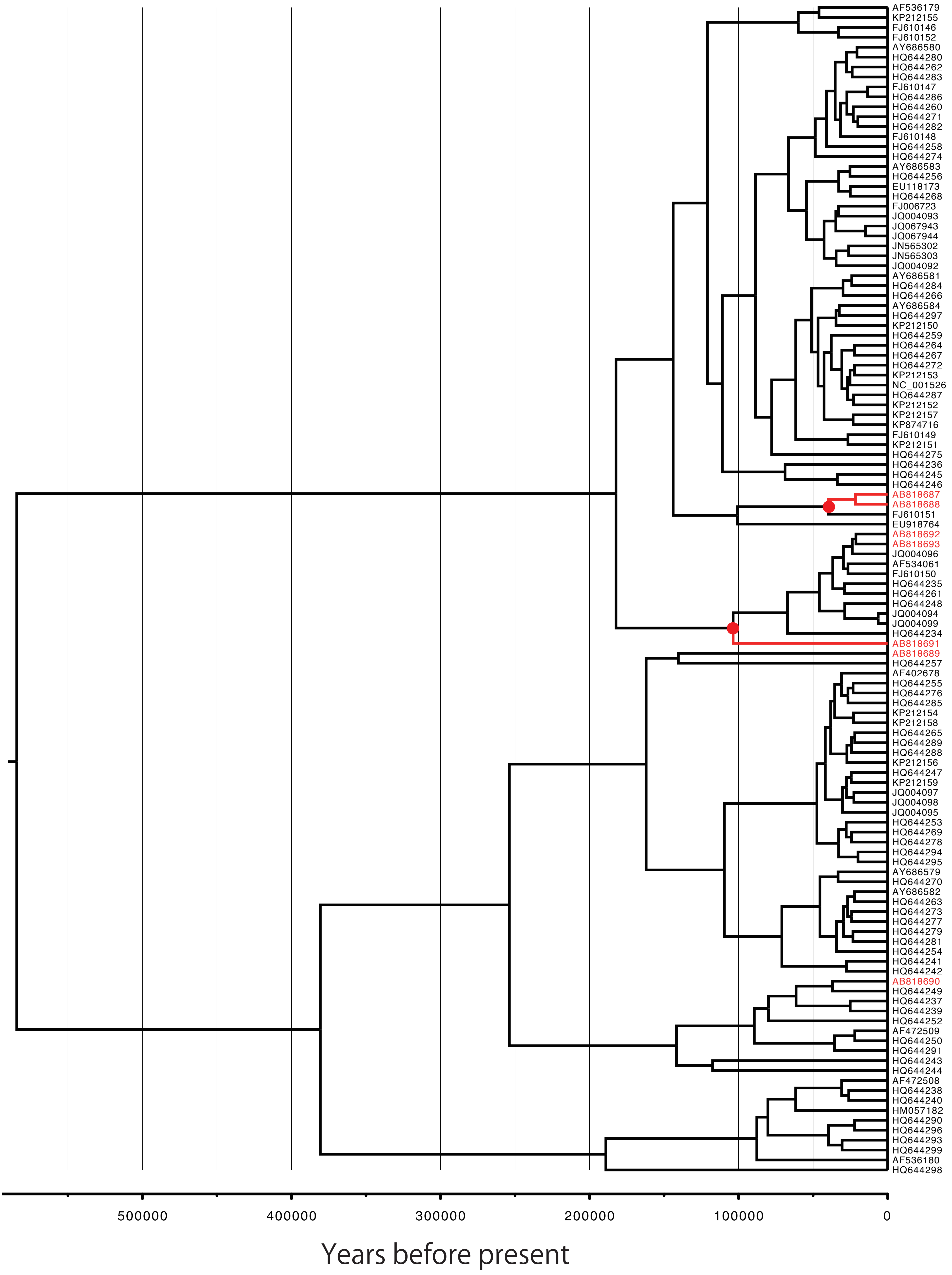

Figure S2. Bayesian MCMC phylogenetic tree of HPV16. 118 HPV16 genome sequences from Pimenoff et al. Time-scaled maximum-clade credibility tree is shown. Red node indicates the position of the most recent common ancestor for the Japan-specific HPV16 variants.
