# Supplementary material for: Ancient Evolutionary History of Human Papillomavirus Type 16, 18 and 58 Variants Prevalent Exclusively in Japan
Source: Viruses. 2022 Feb 24;14(3):464. doi: 10.3390/v14030464 (PMC8953638; doi:10.3390/v14030464)
Supplement: Supplementary file 1 [file viruses-14-00464-s001.zip › Tanaka_viruses_Table S1.pdf]

**Supplementary Table S1.** HPV16 genomic sequences of the A4 sublineage retrieved from Mirabello *et al*.

| Accession number | Accession number |
|---|---|
| MG847626 | MG849049 |
| MG847681 | MG849063 |
| MG847685 | MG849129 |
| MG847704 | MG849203 |
| MG847767 | MG849254 |
| MG847895 | MG849275 |
| MG847922 | MG849304 |
| MG847945 | MG849480 |
| MG847952 | MG849738 |
| MG847985 | MG849739 |
| MG848021 | MG850013 |
| MG848055 | MG850014 |
| MG848130 | MG850069 |
| MG848136 | MG850070 |
| MG848270 | MG850265 |
| MG848387 | MG850361 |
| MG848579 | MG850387 |
| MG848635 | MG850413 |
| MG848768 | MG850586 |
| MG848804 | MG850623 |
| MG849035 | MG850805 |
